# Impact of maintenance dose of eptifibatide in patients with ST-segment elevation myocardial infarction who underwent primary percutaneous coronary intervention

**DOI:** 10.1186/s43044-023-00355-4

**Published:** 2023-04-15

**Authors:** Rozita Jalalian, Babak Bagheri, Jamshid Yazdani Charati, Shahrnaz Khalaghi, Mohammadreza Iranian, Mahsa Mohammadi

**Affiliations:** 1 Department of Cardiology, Faculty of Medicine, Cardiovascular Research Center, Mazandaran University of Medical Sciences, Sari, Iran; 2 Department of Biostatistics, Faculty of Health, Mazandaran University of Medical Sciences, Sari, Iran; 3 Rajaie Cardiovascular Medical and Research Center, School of Medicine, Iran University of Medical Sciences, Tehran, Iran

**Keywords:** ST-segment elevation myocardial infarction (STEMI), Primary percutaneous coronary intervention (PPCI), Eptifibatide

## Abstract

**Background:**

ST-segment elevation myocardial infarction (STEMI) is usually caused by a rupture in the atherosclerotic plaque, followed by platelet aggregation which ultimately leads to acute coronary artery occlusion. So far, few studies have investigated the effect of maintenance dose of Eptifibatide (glycoprotein IIb/IIIa inhibitor) in STEMI patients who underwent primary percutaneous coronary intervention (PPCI). Therefore, in this study, we investigated the effect of maintenance dose of Eptifibatide in patients with STEMI who underwent PPCI. 264 patients who had acute chest pain suggestive of STEMI were entered in the study. All patients received the same dose of bolus dose of Eptifibatide in the cardiac catheterization laboratory. Then the patients were randomly divided into two groups, one group (*n* = 147) received a maintenance dose of intravenous Eptifibatide (infusion of 2 μg/kg/min) and the other group (*n* = 117) did not receive this treatment. Standard medical treatment of STEMI after PPCI was performed based on guidelines and the same in both groups. All patients were evaluated 1, 2, and 3 months after the start of treatment in terms of predicted outcomes.

**Results:**

The occurrence of 3-month major adverse cardiovascular events (MACE) between the case and control groups did not have a statistically significant difference (28.6% versus 35.0%; *P* value: 0.286). Also, investigations showed that the rate of re-infarction (*P* value: 0.024) and target lesion revascularization (*P* value: 0.003) was significantly lower in the group that received Eptifibatide infusion.

**Conclusions:**

Eptifibatide maintenance dose infusion in patients who undergo PPCI in the context of STEMI, does not significantly reduce MACE, although it does significantly reduce re-infarction and target lesion revascularization. It also does not increase the risk of bleeding and cerebrovascular events**.**

## Background

Coronary artery disease is the first and most important death reason in today's societies. What conventionally was called a heart attack, is more precisely called acute coronary syndrome (ACS) nowadays. Patients who go to the emergency room with ACS will divided into Two groups include ST-segment elevation myocardial infarction (STEMI) and non-ST-segment elevation ACS (NSTE-ACS). The latter consist of non–ST elevation myocardial infarction (NSTEMI) and unstable angina (UA) [1, 2].

Each year, approximately 258,000 patients in the United States present to the emergency department with a diagnosis of STEMI, with an incidence of 7.3 per 10,000 [3].

STEMI is usually caused by a rupture or erosion in the atherosclerotic plaque, followed by platelet aggregation and thrombosis which ultimately leads to acute coronary artery occlusion and myocardial damage. The preferred treatment strategy includes emergency reperfusion through primary percutaneous coronary intervention (PPCI) [4].

Of course, despite reperfusion of blood flow in the coronary arteries that caused the infarct, there is myocardial perfusion disorder in some patients who have undergone successful PCI [4, 5]. One of the main causes of myocardial reperfusion disorder is the embolization of thrombotic substances including platelet aggregations in the distal microcirculation. Widespread use of PCI may also create a thrombotic condition due to wounding the vessel wall and stimulate platelet activation and proliferation of new intima [6].

The first step to initiate arterial thrombosis is endothelial damage and exposure of subendothelial matrix glycoprotein (GP) to circulating platelets, followed by platelet adhesion [7]. GP IIb/IIIa are the most abundant integrins on the surface of platelets. Ligands such as fibrinogen bind to GP IIb/IIIa of adjacent platelets and lead to platelet aggregation and thrombus formation [8].

In recent years, the implementation of adjunctive mechanical and pharmacological therapies during PPCI has significantly improved clinical outcomes in STEMI patients. Among these treatments, aspirin and P2Y12 inhibitors can be mentioned, which play an essential role in the medical treatment of STEMI patients [9, 10].

Among other treatments, the use of GP IIb/IIIa inhibitors can be mentioned, which have significantly reduced the incidence of distal embolization and improved reperfusion of capillary blood flow and clinical outcomes in STEMI patients. However, this approach may also have disadvantages and may be associated with an increased risk of bleeding [11–14].

Three intravenous drugs of GP IIb/IIIa inhibitors namely Abciximab, Eptifibatide and Tirofiban are widely used in PCI and in the treatment of ACS. Eptifibatide binds to GP IIb/IIIa receptors and prevents the binding of fibrinogen to receptor and causes an anti-platelet effect. On the other hand, inhibition of GP IIb/IIIa reduces the activation of prothrombin replication factors. Therefore, inhibition of GP IIb/IIIa may have both antiplatelet and anticoagulant effects [7, 8, 15, 16].

According to European and American guidelines, intravenous injection of GP IIb/IIIa inhibitors in patients with STEMI who underwent PPCI and have evidence of high thrombosis or no-reflow phenomenon seems reasonable (Class: IIa, LoE: C) [9, 10]. However, so far few studies (Low Level of Evidence) have investigated the effect of maintenance dose of Eptifibatide in STEMI patients who underwent PPCI. Therefore, in this study, we investigated the effect of maintenance dose of Eptifibatide in patients with STEMI who underwent PPCI.

## Methods

### Patient selection

The study population is patients older than 18 years old who visited between September 2017 and August 2018 at Fatemeh Zahra hospital of Sari, Northern Iran and underwent PPCI with STEMI. The people in the study were patients who had a history of chest pain and an ECG suggestive of STEMI (new ST elevation in two contiguous leads by 0.1 mV or more) from 30 min before admission to 12 h before hospital admission.

Patients who presented with cardiogenic shock, patients who became candidates for PCI after receiving fibrinolytics, patients who became candidates for emergent coronary artery bypass graft surgery after angiography, patients under 18 years old or over 80 years old, pregnant patients, patients with kidney or liver failure and patients who had contraindications for Eptifibatide use (active internal bleeding, brain neoplasm or aneurysm, stroke in the last 2 years, intracerebral trauma or surgery in the last 2 months, uncontrolled high blood pressure, and thrombocytopenia) were excluded from the study.

### Study design and ethics

**Fig. 1:**
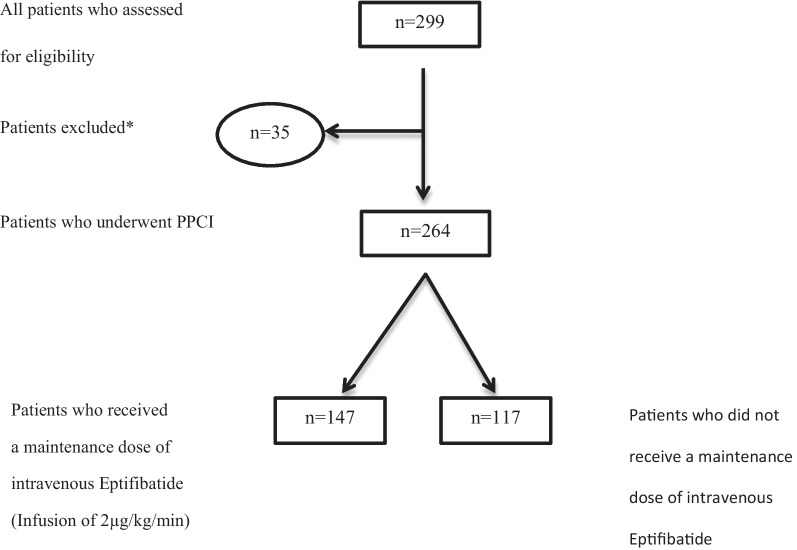
Clinical trial flowchart. *Patients who presented with cardiogenic shock, patients who became candidates for PCI after receiving fibrinolytics, patients who became candidates for emergent coronary artery bypass graft surgery after angiography, patients under 18 years old or over 80 years old, pregnant patients, patients with kidney or liver failure and patients who had contraindications for Eptifibatide use (see main text, methods, patient selection) were excluded from the study. PPCI: Primary Percutaneous Coronary Intervention

This study was a prospective, double-blind, randomized controlled clinical trial that was conducted on 264 patients with STEMI who underwent PPCI. The sample size of this study was (calculated based on statistical formulas and after 10% downfall), 264 people. All these patients were treated similarly with aspirin (300 mg), clopidogrel (600 mg) and statin after electrocardiographic confirmation of STEMI. Then all patients underwent PPCI with Door-to-Device (DTD) time less than 90 min. DTD time was defined as the time from arrival to the facility to time of catheterization device deployment, which should be 90 min or less. Regarding the angiography method, femoral approach was preferred. All patients received the same dose of intravenous heparin and bolus dose of Eptifibatide (180 μg/kg/10 min) in the cardiac catheterization laboratory. Then the patients were randomly divided into two groups, one group (*n* = 147) received a maintenance dose of intravenous Eptifibatide (infusion of 2 μg/kg/min) and the other group (*n* = 117) did not receive this treatment. Study design has been summarized in Fig. 1. Randomization was done by sealed envelopes at the end of the procedure. Administering the maintenance dose of eptifibatide started during the first hour after the bolus dose injection and continued for 12 h. Standard medical treatment of ACS after PCI was performed based on guidelines and the same in both groups with aspirin (80 mg), clopidogrel (75 mg), lipid-lowering drugs, beta-blockers and angiotensin-converting enzyme inhibitors (ACEIs) or angiotensin receptor blockers (ARBs) [9, 10]. All patients were re-evaluated 1, 2, and 3 months after the start of treatment in terms of predicted outcomes.

Also, the objectives of this research project and its implementation method were explained to all patients, and they entered this project after obtaining oral and written informed consent. It should be noted that this study was registered and approved (Approval Number: 1394050) by the Ethics Committee of Mazandaran University of Medical Sciences before its implementation.

### Primary and secondary outcomes

The primary outcome in this study was major adverse cardiovascular events (MACE), a composite of cardiovascular death, reinfarction, cerebrovascular events, major and minor bleeding, target vessel revascularization (TVR), and target lesion revascularization (TLR).

Major bleeding based on the standardized definitions of bleeding for cardiovascular clinical trials includes: fatal bleeding that leads to death within 7 days, intracranial or retroperitoneal bleeding, bleeding that is accompanied by a drop of at least 3–5 units of hemoglobin or need to inject at least 2–4 units of blood, bleedings that lead to hemodynamic disorders and require intervention, access site bleedings that require intervention, and hematoma with a size of 5 cm or more [17]. Bleedings smaller than those mentioned above were considered as minor bleeding.

TVR was defined as unplanned repeat PCI or bypass graft placement for a stenosis in another part of the vessel treated at the index PCI. TLR was defined as repeat PCI or bypass graft placement for restenosis at the lesion treated during index PCI or occurring within 5 mm of the PCI site [18].

Secondary outcomes include separate examination of in-hospital and out-of-hospital death due to cardiovascular diseases, re-infarction, cerebrovascular events, major bleeding, minor bleeding, TVR and TLR.

### Statistical analysis

The obtained data were entered into SPSS software (Statistical Package for the Social Sciences) version 24. First, they were summarized using methods based on descriptive statistics, including the mean ± standard deviation for quantitative variables (such as age) and frequency tables for qualitative variables, and then to compare the groups in terms of background variables, using the t-test, Chi-square and finally logistic regression were analyzed. *P* value less than 0.05 was considered significant.

## Results

### Study population

Demographic and clinical characteristics of all patients are shown in Table 1. There was no significant difference between the two groups in terms of gender and age. Among the patients participating in the study, 147 people were in the intervention group, 95 of them were men (64.6%) and 52 were women (35.4%), and their average age was 59.85. Also, out of 117 patients participating in the control group, 65 were men (55.6%) and 52 were women (44.4%), whose average age was 61.78. In terms of coronary artery disease risk factors (diabetes mellitus, hypertension, hyperlipidemia and cigarette smoking) there was no significant difference between the two groups. Also, out of the total of 147 patients studied in the intervention group, the location of myocardial infarction in 56.5% of cases was in the anterior regions, 39.3% in the lower regions and in 4.6% in the lateral regions. In the control group, the location of myocardial infarction in 45.3% of cases was in the anterior regions, in 52.1% in the lower regions and in 2.6% in the lateral regions, and also in this sense, no significant difference was observed between the two studied groups.

**Table 1:** Patients’ demographics and clinical characteristics

	Case group (%)	Control group (%)	*P* value
Sex			
Male	95 (64.6)	65 (55.6)	0.16
Female	52 (35.4)	52 (44.4)	
Age			
< 60 years	79 (53.7)	59 (50.4)	0.62
> 60 years	68 (46.3)	58 (49.6)	
Diabetes mellitus	41 (27.9)	34 (29.1)	0.89
Hypertension	52 (35.4)	52 (44.4)	0.16
Hyperlipidemia	42 (28.6)	44 (37.6)	0.14
Cigarette smoking	41 (27.9)	31 (26.5)	0.12
Location of myocardial infarction			0.12
Anterior territory	83 (56.5)	53 (45.3)	
Inferior territory	58 (39.3)	61 (52.1)	
Lateral territory	6 (4.1)	3 (2.6)	

### Primary outcomes

**Fig. 2:**
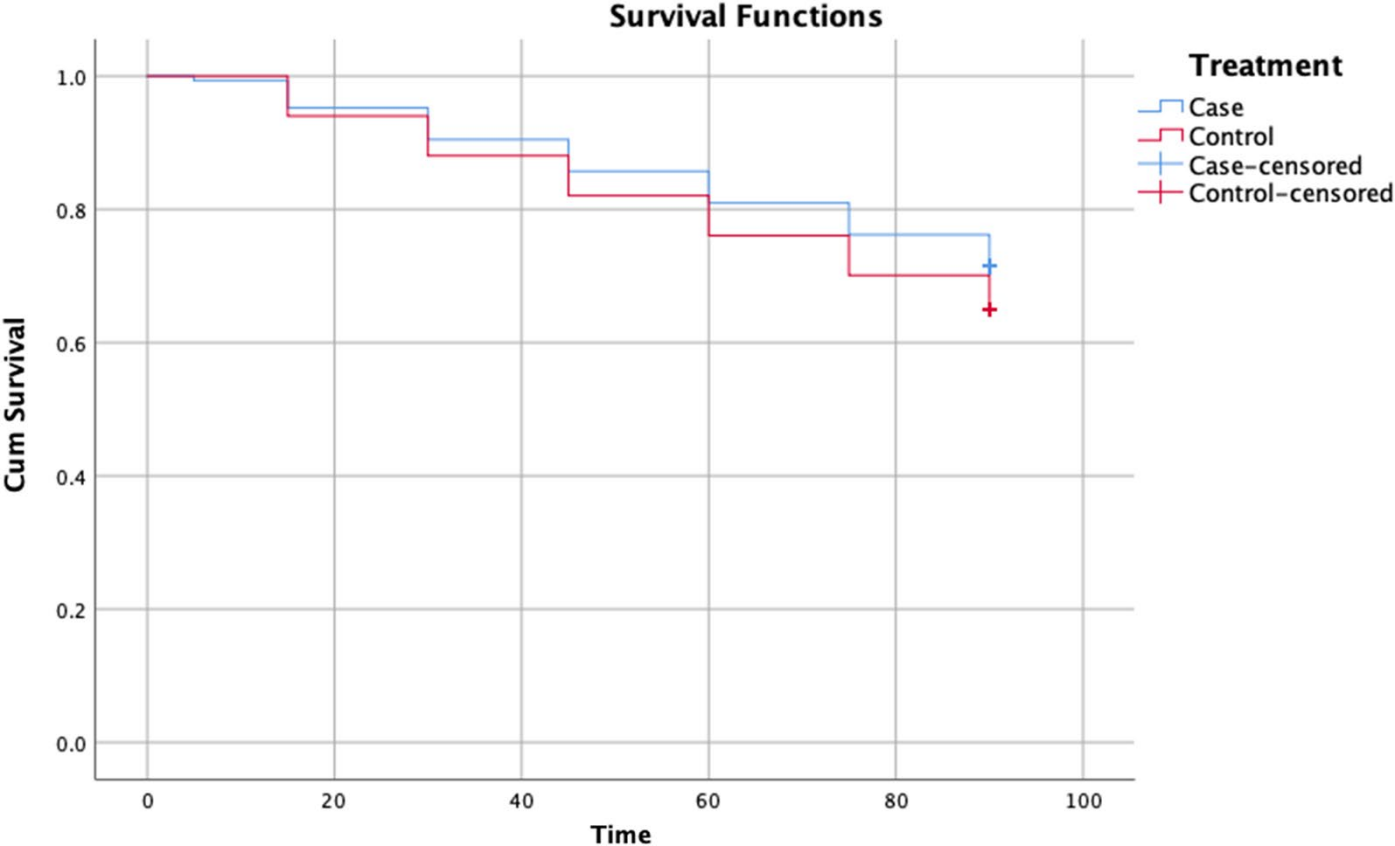
Kaplan–Meier curves for survival free of major adverse cardiovascular events (MACE) in case and control groups

The studied patients were followed up for 3 months after PPCI. Considering the low mortality rate in STEMI patients who undergo PPCI, in order to investigate the effect of Eptifibatide drug on the consequences and cardiovascular complications, the patients participating in this study in terms of MACE, which includes a combination of death due to cardiovascular diseases, re-infarction, cerebrovascular events, major and minor bleeding, target vessel revascularization (TVR) and target lesion revascularization (TLR) were divided into two groups (Fig. 2): the group that experienced MACE and the group that did not experience MACE. The results of our study showed that the occurrence of 3-month MACE between the case and control groups did not have a statistically significant difference (42 patients equivalent to 28.6% in the case group versus 41 patients equivalent to 35% in the control group), which means that receiving a maintenance dose of Eptifibatide did not significantly reduce the combination of mortality and morbidity in the patients (*P* value: 0.286).

Also, the 3-month MACE rate in the case and control groups was compared in different subgroups (Table 2). These findings showed that in our studied patients, the relationship between the occurrence or non-occurrence of MACE after Eptifibatide infusion with hypertension and hyperlipidemia is significant. This means that in patients with hypertension and hyperlipidemia, Eptifibatide infusion has significantly reduced mortality and morbidity. Of course, this relationship was not seen in diabetic patients and smokers.

**Table 2:** The 3-month MACE rate in different subgroups

	MACE −	MACE +	*P* value
Diabetes mellitus	53 (29.3%)	22 (26.5%)	0.64
Hypertension	64 (35.3%)	40 (48.2%)	0.04
Hyperlipidemia	48 (26.5%)	38 (45.8%)	0.02
Cigarette smoking	48 (26.5%)	24 (28.9%)	0.68

*MACE* major adverse cardiovascular events

### Secondary outcomes

Three months after PPCI, secondary outcomes including in-hospital and out-of-hospital death due to cardiovascular diseases, re-infarction, cerebrovascular events, major bleeding, minor bleeding, TVR and TLR were investigated (Table 3). Investigations showed that the rate of re-infarction and performing TLR was significantly lower in the group that received Eptifibatide infusion, while there was no significant difference of death due to cardiovascular diseases, cerebrovascular events, major and minor bleeding and TVR between two groups.

**Table 3:** Secondary outcomes after primary percutaneous coronary intervention in case and control groups

	Case group (%)	Control group (%)	*P* value
In-hospital death due to cardiovascular disease	2 (1.4)	2 (1.7)	0.92
Out of hospital death due to cardiovascular disease	6 (4.1)	2 (1.7)	0.30
Re-infarction	1 (0.7)	7 (6)	0.02
Target Lesion Revascularization	2 (1.4)	11 (9.4)	0.003
Target Vessel Revascularization	15 (10.2)	16 (13.7)	0.10
Cerebrovascular events	3 (2)	0 (0)	0.25
Major bleeding	6 (4.1)	2 (1.7)	0.30
Minor bleeding	4 (2.7)	2 (1.7)	0.69

## Discussion

Our study showed that in STEMI patients who underwent PPCI, although Eptifibatide maintenance dose infusion, causes a significant reduction in re-infarction and TLR, it does not cause a significant reduction in MACE.

The most studied GP IIb/IIIa inhibitor in patients with STEMI who undergo PPCI is Abciximab [8]. Although previous large studies showed that administration of Abciximab in patients undergoing PPCI due to STEMI reduces death, re-infarction and ischemic events [19, 20], subsequent studies showed that its administration especially in patients whom receive thienopyridine drugs at the same time, does not significantly reduce death and re-infarction [21, 22]. However, the results of our study showed that the administration of Eptifibatide in a subgroup of patients with hypertension and hyperlipidemia reduces MACE. Whether this effect of the drug is related to the primary antiplatelet effect of Eptifibatide or the non-specific anti-inflammatory properties of it is not determined by our study.

In 2010, Zeymer and colleagues in the EVA-AMI Trial showed that Eptifibatide infusion, as an adjunct to PPCI in the treatment of STEMI, is as effective as Abciximab, and even the rate of re-infarction was significantly lower with Eptifibatide, while There was no significant difference of the bleeding rate between the two groups [23]. This finding is consistent with the lower rate of re-infarction in our intervention group.

So far, many studies have been published on the adjuvant effect of Eptifibatide infusion in patients undergoing PCI. One of the most important studies is the ESPRIT Trial. The findings of this study showed that the administration of Eptifibatide leads to a significant reduction of 48-h and 30-day MACE (combination of death, myocardial infarction and TVR) [24]. The inconsistency of the results of our study with ESPRIT Trial can be due to the smaller sample size of our study (264 patients vs. 2064 patients) and also the shorter infusion time of this drug in our study (12 h vs. 18–24 h) and as a result receiving a lower dose of the drug. GP IIb/IIIa inhibitors block the binding of circulating fibrinogen and von Willebrand factor to the GP IIb/IIIa receptor thus preventing the cross-linking of platelets necessary for aggregation and thrombosis. These agents have also been shown to disaggregate freshly formed, platelet-rich thrombi in a “dose-dependent manner” ranging from no disaggregation at low doses up to significant disaggregation at clinically relevant doses [25].

Most of the studies conducted on Eptifibatide are on patients who are candidates for PCI in the context of NSTE ACS. To our knowledge, few clinical trials have been conducted on the effect of Eptifibatide in patients undergoing PPCI due to STEMI. A retrospective study that was conducted between 2000 and 2009 in the United States and showed that the administration of Eptifibatide reduces mortality in STEMI patients who undergo PPCI [25], while the study by Le May et al., and the study of Shariati et al., showed that the administration of this drug does not reduce MACE (combined mortality and recurrent myocardial infarction) [13, 26]. These observations, however, have not been fully supported by a retrospective analysis of clinical outcomes in the ESPRIT trial, accepting that this was not a primary PCI study and not everyone in the preloading group received the 600 mg dose of Clopidogrel, the study demonstrated that the efficacy of Eptifibatide was maintained irrespective of Thienopyridine preloading.

In a systematic review and meta-analysis in 2019, Karatanos et al. showed that routine treatment with GP IIb/IIIa inhibitor in STEMI reduces mortality and re-infarction at 30 and 60 days [27]. However, most of the studies used in this review article were about Abciximab and Tirofiban, and there was only one study about the administration of Eptifibatide, in which there was no significant difference between the group receiving the drug and the control group in the primary outcome (which was the combination of 30-day mortality and re-infarction) [26, 27].

The present study showed that Eptifibatide maintenance dose infusion decreases TLR. TLR was defined as repeat PCI for restenosis at the lesion treated during index PCI. Although this finding is not consistent with the study of Shariati et al. [13], it can be justified considering the mechanism of effect of Eptifibatide. Eptifibatide prevents the binding of fibrinogen to receptor and reduces the activation of prothrombin replication factors.

Also, our study showed that Eptifibatide administration does not increase major and minor bleeding and cerebrovascular events. One of the reasons for the low rate of bleeding in the present study is the careful selection of patients based on the inclusion and exclusion criteria, which reduces the chance of bleeding. The homogeneity of the two groups in terms of the amount of major and minor bleeding shows that the possibility of bleeding complications should not prevent the administration of this drug infusion in high-risk patients.

### Study limitations

Some limitations of our study must be declared. The relatively small sample size, short follow-up period and “single-center” randomized clinical trial were some of the limitations of the study. In future studies in STEMI patients (an area where the effect of Eptifibatide has not been sufficiently investigated), with a larger sample size and with a higher power, its generalizability to the community and its validity can be improved.

## Conclusions

Eptifibatide maintenance dose infusion in patients who undergo PPCI in the context of STEMI, does not significantly reduce MACE, although it does significantly reduce re-infarction and TLR. It also does not increase the risk of bleeding and cerebrovascular events.

## Data Availability

Data available on request from the authors. The data that support the findings of this study are available from the corresponding author upon reasonable request.

## Abbreviations

STEMI: ST-segment elevation myocardial infarction
PPCI: Primary percutaneous coronary intervention
MACEs: Major adverse cardiovascular events
ACS: Acute coronary syndromes
NSTE-ACS: Non-ST-segment elevation acute coronary syndromes
ACEIs: Angiotensin-converting enzyme inhibitors
ARBs: Angiotensin receptor blockers
TVR: Target vessel revascularization
TLR: Target lesion revascularization
